# Blocking hepatic metastases of colon cancer cells using an shRNA against Rac1 delivered by activatable cell-penetrating peptide

**DOI:** 10.18632/oncotarget.12854

**Published:** 2016-10-24

**Authors:** Ying Bao, Huihui Guo, Yongliang Lu, Wenming Feng, Xinrong Sun, Chengwu Tang, Xiang Wang, Mo Shen

**Affiliations:** ^1^ Department of Surgery, First Affiliated Hospital, Huzhou University, The First People's Hospital of Huzhou, Huzhou, 313000, China; ^2^ Department of Medicine, Huzhou University, Huzhou, 313000, China; ^3^ Department of Laboratory Medicine, The First Affiliated Hospital of Wenzhou Medical University, Wenzhou, 325000, China

**Keywords:** cell penetrating peptide, Rac1, metastasis, adhesion, tumor targeting nanoparticle

## Abstract

Hepatic metastasis is one of the critical progressions of colon cancer. Blocking this process is key to prolonging survival time in cancer patients. Studies on activatable cell-penetrating peptides (dtACPPs) have demonstrated their potential as gene carriers. It showed high tumor cell-targeting specificity and transfection efficiency and low cytotoxicity in the *in vitro* settings of drug delivery. However, using this system to silence target genes to inhibit metastasis in colorectal cancer cells has not been widely reported and requires further investigation. In this study, we observed that expression of Rac1, a key molecule for cytoskeletal reorganization, was higher in hepatic metastatic tumor tissue compared with prime colon cancer tissue and that patients with high Rac1-expressing colon cancer showed shorter survival time. Base on these findings, we created dtACPP-PEG-DGL (dtACPPD)/shRac1 nanoparticles and demonstrated that they downregulated Rac1 expression in colon cancer cells. Moreover, we observed inhibitory effects on migration, invasion and adhesion in HCT116 colorectal cancer cells *in vitro*, and our results showed that Rac1 regulated colon cancer cell matrix adhesion through the regulation of cytofilament dynamics. Moreover, mechanically, repression of Rac1 inhibiting cells migration and invasion by enhancing cell to cell adhesion and reducing cell to extracellular matrix adhesion. Furthermore, when atCDPPD/shRac1 nanoparticles were administered intravenously to a HCT116 xenograft model, significant tumor metastasis to the liver was inhibited. Our results suggest that atCDPP/shRac1 nanoparticles may enable the blockade of hepatic metastasis in colon cancer.

## BACKGROUND

Colorectal cancer ranks the third most common cancer in the world. Metastasis is the major course of cancer mortality. Liver metastases are very common in people with advanced colon cancer [1], which is the major cause of death, and the prognosis of these patients was so poor that it was uncertain whether any treatment should be recommended for their primary cancer. Some patients received chemotherapy simply for the sake of doing something in regards to treatment, despite little if any benefit for the patient [2].

The metastatic process is complicated, including invasion of local tumor cells, entering into the blood and lymphatic circulation, as well as colonizing into distal organs, which requires collaborations from a variety of molecules, such as E-cadherin, Rho GTPases, and metalloprotease (MMPs) [3, 4].

Rac1 belongs to the Rho GTPase family, which is thought to be involved in the regulation of actin dynamics [5]. Rac1 is responsible for the formation of lamellipodia at the leading edge [6] and affects cell motility by regulating cytoskeletal reorganization [7]. Ji found that Rac1 was related to invasiveness and is a promising therapeutic target for treatment of gastric cancer [8]. Overexpression and overactivation of Rac1 resulted in increased cell proliferative and metastatic capacities in gastric cancer cells. A Rac1 inhibitor, which inhibited Rac1 activity, abrogated cell migration in head and neck squamous cell carcinoma cells (HNSCC) [9].

RNA interference (RNAi), a potential technique in treatment of cancer, can suppress the expression of target genes and improve the outcome of chemotherapeutics [10]. Successful inhibition of Rac1 expression using RNAi leads to decrease of blood vessel density and slower of tumor growth [11]. Many materials have been developed to deliver shRNA vectors *in vivo*. However, how to target the tumors specifically is a remaining barrier.

MMPs, secret by cancer cells [12], are required to digest molecules of cell adhesion including E-cadherin and degrade ECM proteins and are therefore important for the migration and invasion of cancer cells. Huang et al. developed an activatable cell-penetrating peptide (dtACPP) that is dual-activated by the specific tumor microenvironment, including reduced pH (pH 5.8-7.2) and overexpressed MMP2 [13]. In this system, via R-malemidyl-ω-N-hydroxysuccinimidyl polyethyleneglycol (MAL-PEG-NHS), dtACPP was conjugated to the surface of poly-L-lysine (DGL), which has been used to deliver gene into tumor cells, due to its ability to encapsulate DNA to form nanoparticles, which provides a tool to target specific genes in tumor cells [13], and shows the EPR (enhanced permeability and retention) effect. Although this system showed a promising efficacy in the induction of anti-angiogenesis and apoptosis, it did not show an anti-invasion effect in colon cancer cells.

In this study, we employed dtACPPD/shRac1 nanoparticles, evaluated their therapeutic efficacy *in vitro* and *in vivo*, and investigated the underlying mechanisms both *in vitro* and *in vivo*.

## RESULTS

### Rac1 is related to the metastasis and survival time of colon cancer

**Figure 1:**
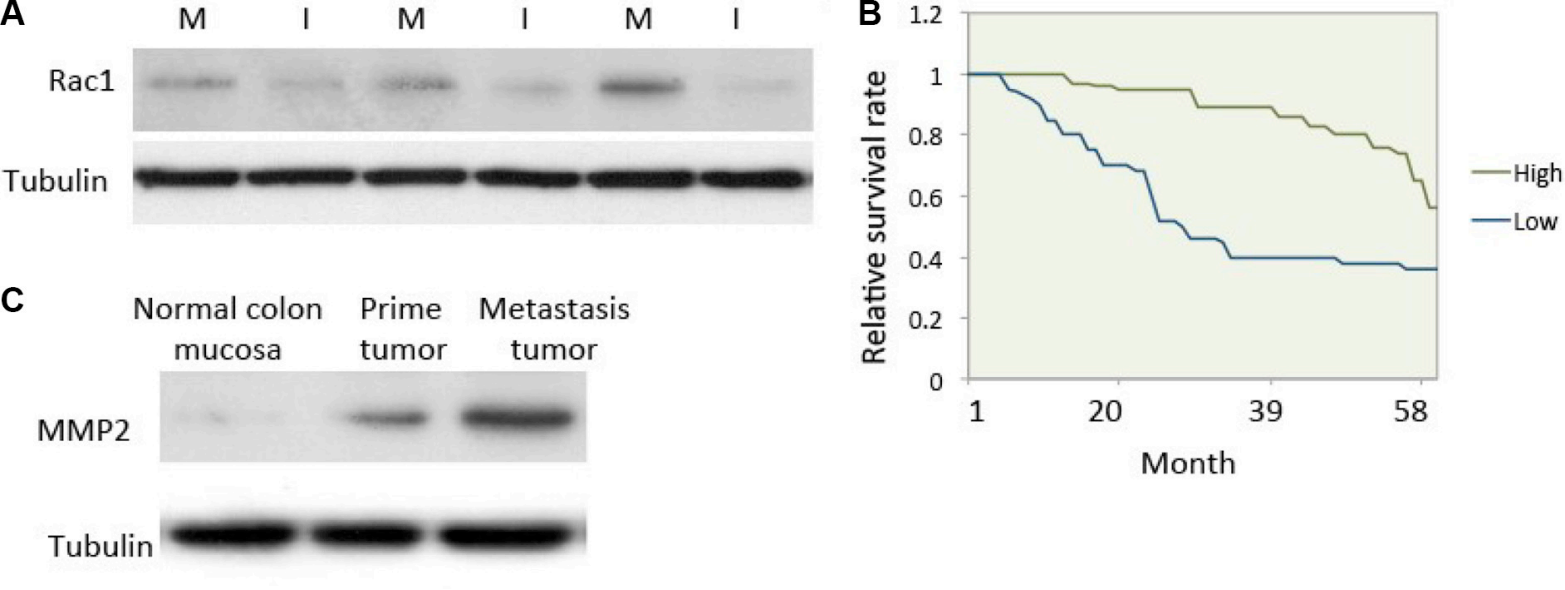
Rac1 expression was elevated in human metastatic colon cancer (**A**) Expression of Rac1 in human colon cancer tissue. Human colon cancer tissue was collected and western blotting was used to test the expression of Rac1. Equal amounts of cell lysate from *in situ* cancer tissue (lanes I) and hepatic metastasis tissue (lanes M) from patients. (**B**) Survival curves for colon cancer patients with either high or low Rac1 expression (*P* < 0.01). (**C**) Expression of MMP2 in normal colon mucosa, prime colon cancer tissue and metastasis cancer tissue in patients. Protein was extracted from patients and subjected to western blot.

Rac1 is a key molecule that regulates cell motility [7]. Rac1 expression and activity are both enhanced in colon cancer and could speed up the metastasis of cancer cells [18]. In this study, we compared Rac1 expression in *in situ* colon cancer tissue to hepatic metastatic tumor tissue. The detail information for 66 patients was showed in Table 1. The results showed that Rac1 expression in metastatic tumor tissue was much higher than in prime cancer tissue (Figure 1A). A 5-year follow-up after surgery and/or chemotherapy suggests that patients with high Rac1 levels in their tumors have a shorter survival than those with tumors with low Rac1 levels (Figure 1B). MMP2 was highly express in cancer cells to enable cells to break down surrounding tissue for the invasive behavior [19]. We therefore compared MMP2 expression in normal colon mucosa, prime colon cancer tissue and metastasis cancer tissue. MMP2 expression was hardly observed in normal colon mucosa, but markedly high in prime colon cancer and much higher in metastasis cancer tissue in colon cancer patients (Figure 1C).

**Table 1:** Detailed information regarding the 66 colon patient specimens

Detailed information	No. of tumor specimens (*N* = 66)
Gender	
Male	42
Female	20
Age (years)	
> 60	45
≤ 60	11
Grade	
Well differentiated	9
Moderately differentiated	29
Poorly differentiated	28
Depth of tumor invasion	
Mucous layer	1
Muscular layer	9
Serous layer	56

### Preparation and characterization of dtACPPD/ shRac1 nanoparticles

In the tumor microenvironment, the overexpressing MMP2 and reduced pH were commonly combined used to improve the tumor targeting and cellular internalization [19]. The dtACPPD nanoparticle system was developed and identified as it is triggered by the tumor microenvironment.^13^ Therefore, in this study, we employed the dtACPPD/ shRac1 system and evaluated its anti-metastatic capacity in colorectal cancer cells.

**Figure 2:**
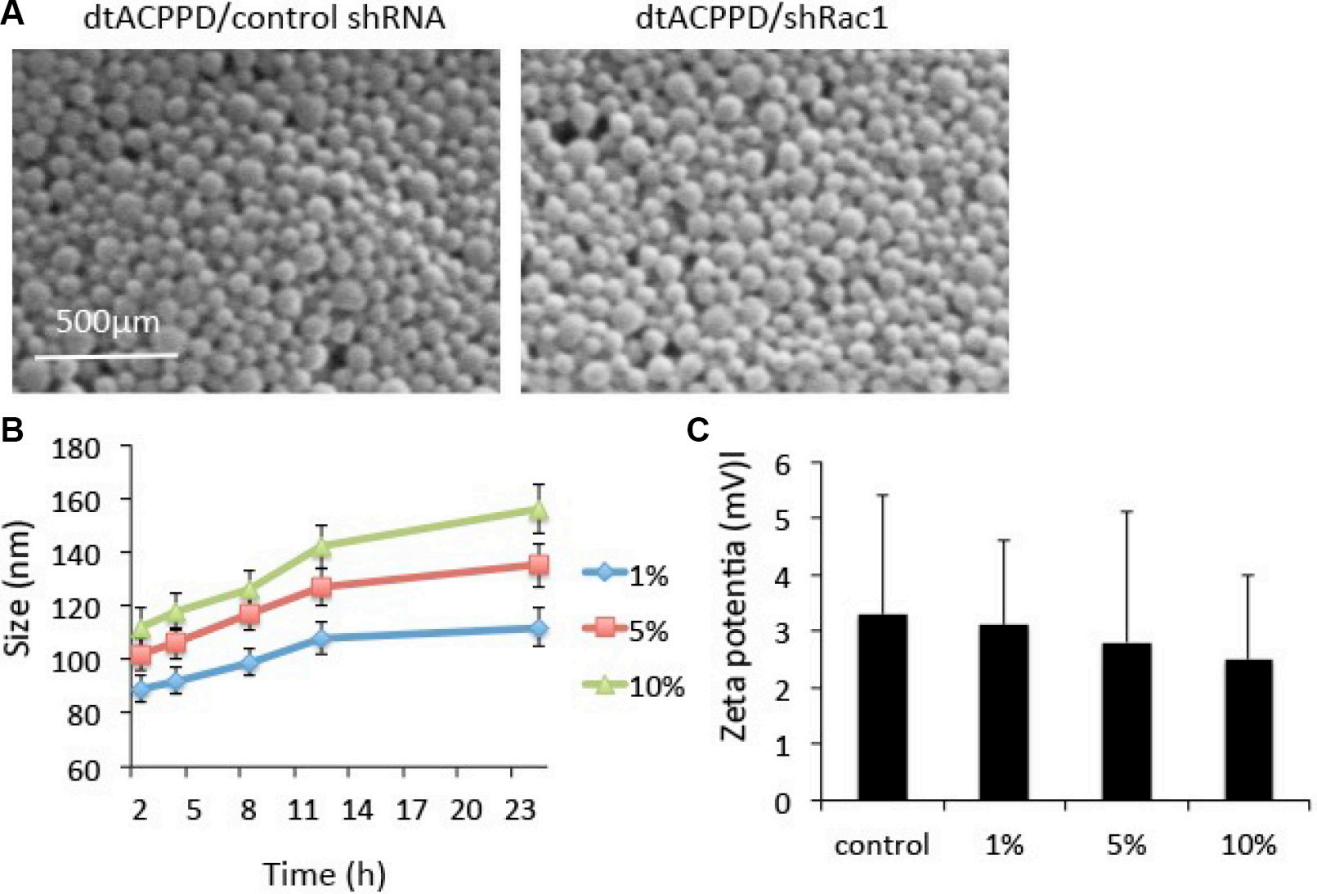
*In vitro* characterization of dtCDDP/shRNA (**A**). Scanning electron micrographs of dtCDDP/ control shRNA NPs and dtCDDP/shRac1 NPs. (**B**). The influence of serum concentration on the size of NPs. (**C**). The influence of serum concentration on Zeta potential.

Cationic polymer nonviral vectors dtACPPD were constructed according to a previous report by Huang et al. 2013. Scanning electron microscopy (SEM) images showed that the dtACPPD/shRac1 particles were analogous spherical shapes (Figure 2A). The size of dtACPPD/shRac1 particles was 113.6 ± 2.9 nm with a narrow distribution. This size range was suitable for tumor-targeting delivery because the size was able to perform the EPR effect and prolong the existence of blood circulation by not only penetrating into the tumor tissue and avoiding reticuloendothelial system (RES)-mediated clearance, but also reducing renal filtration. The zeta-potential value was 2.1 ± 0.7 mV.

The *in vitro* stability of the dtACPPD/shRac1 particles was evaluated in the presence of 1%, 5% and 10% bovine serum albumin (BSA). Particles were suspended in a series of concentrations of BSA at 37°C for different durations of time. The particles were enlarged when the BSA concentrations were increased and incubation time was prolonged (Figure 2B). The particle size did not change significantly within 24 h of incubation, indicating the particles were stable in 1% BSA. However, the size of particles increased when incubated with 5% and 10% BSA. However, the particles showed good dispersibility. In addition, the zeta potential of particles incubated with different concentrations of BSA was constant (Figure 2C).

### Cellular uptake study and knock down efficacy of dtACPPD/ shRac1 nanoparticles

**Figure 3:**
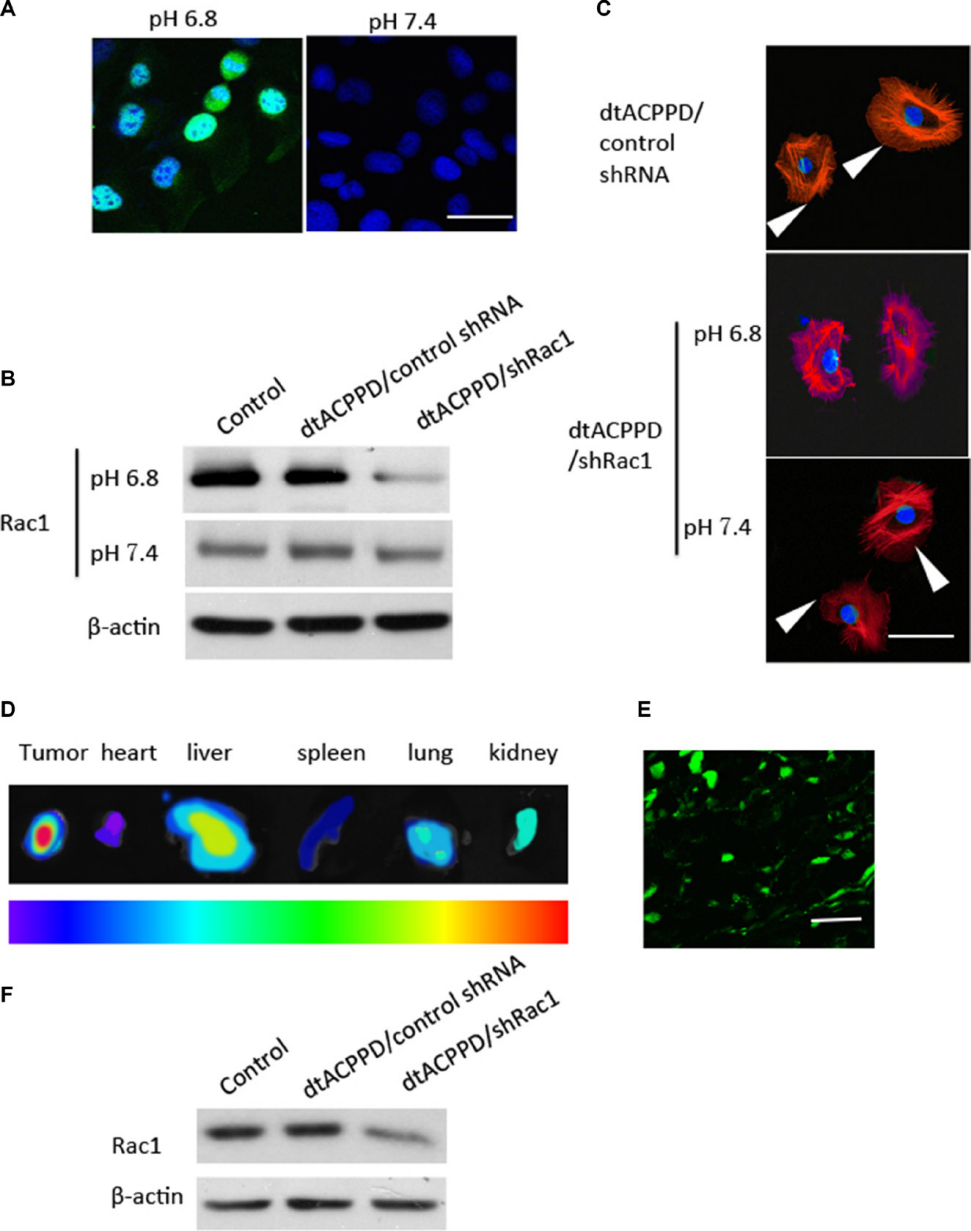
Penetration of dtACPPD/shRNA nanoparticles in colon cancer cells *in vitro* and *in vivo* (**A**). Florescence image of colorectal cancer cells treated with dtACPPD/shRNA nanoparticles under pH 7.4 or 6.8. Scale bar is 50 μm. (**B**). Expression of Rac1 after penetration of dtACPPD/shRNA nanoparticles as tested by western blot. (**C**) Cytoskeletal organization was detected by immunofluorescent staining using an antibody against phalloidin (red). DAPI was used to stain nuclei. Arrows showed the lamellipodias. Scale bar is 20 μm. (**D**) Tumor bearing mice were treated with dtACPPD/shRNA nanoparticles. Fluorescence imaging showed the *in vivo* distribution. The corresponding exposed main organs were excised at 24 h after administration. The intensity of the signal dark red is the strongest, while dark blue is the weakest, as shown by the bar. (**E**) Tumor sections showed the accumulation of DNA at the interior of the tumors (Green). Scale bar is 100 μm. (**F**) Proteins of tumors with different treatments were lysed and Rac1 expression was detected by western blot.

The cellular uptake study was used to measure the efficacy of *in vitro* internalization. The shRNA against Rac1 was constructed with the enhanced green fluorescence (EGFP) gene. The efficacy of dtACPPD/shRac1 delivery was then studied in HCT116 cells at pH 7.4 or pH 6.8. Figure 3A shows green fluorescence in the nucleus of cells with incubation of dtACPPD/shRac1 at pH 6.8, indicating the Rac1 shRNA was integrated into the cellular genome after incubation of dtACPPD/shRac1 in an acidic environment for 24 h, suggesting the acid-sensitive ability of the dtACPPD structure. Furthermore, the level of Rac1 expression was analyzed by western blot, which is shown in Figure 3B. The dtACPPD/shRac1 nanoparticles showed significant silencing efficiency of Rac1 expression at pH 6.8 (89.2% down-regulation, *p* < 0.01) compared to that at pH 7.4 (5.6% down-regulation) (Figure 3B). As Rac1 is a key molecule in the regulation of cytoskeletal reorganization, we assessed the cytoskeleton of HCT-116 cells after exposure to dtACPPD/shRac1 nanoparticles for 72 h. Immunofluorescent staining showed a disorganized cytoskeleton when cells were treated with nanoparticles at pH 6.8 following the down-regulation of Rac1; meanwhile, the cytoskeleton did not show obvious alterations at pH 7.4 (Figure 3C). The results suggested that dtACPPD/shRac1 showed good tumor target ability and internalization efficacy.

### *In vivo* biodistribution of dtACPPD/ shRac1 nanoparticles

The *in vivo* biodistribution, tumor-targeting characteristics and Rac1 silencing efficacy of dtACPPD/shRac1 were investigated in an HCT-116 cell tumor model in BALB/c nude mice. Figure 3 showed that, forty-eight hours after intravenous administration, the distribution of EGFP labeled Rac1 shRNA showed very high accumulation in the tumors, benefiting from the en- hanced permeability and retention (EPR) effect (Figure 3D). Furthermore, within the tumor, expression of EGFP was observed in the tumor cells, indicating the successful intratumoral injection of particles and gene transfection (Figure 3E). These results suggested that dtACPPD/shRac1 nanoparticles can accumulate in tumors, benefiting from the two triggers, reduced pH and overexpressed MMP2. When dtACPP is activated prematurely to expose CPP in the circulation, nanoparticles accumulated instantly in the main metabolic and highly perfused organs to perform the EPR effect [13]; therefore, the particles was found distributed in the liver, kidney and lung.

After systemic administration, tumors were removed and protein was extracted from tumors. Western blot analysis showed that Rac1 was down-regulated in tumors treated with dtACPPD/shRac1 (76.4% down-regulation, *p* < 0.01) (Figure 3F), suggesting the ideal tumor-targeting ability of the dtACPPD/shRac1nanoparticles.

### *In vitro* cell migration, polarity and invasion of colon cancer cells after treatment with dtACPPD/ shRac1 nanoparticles

**Figure 4:**
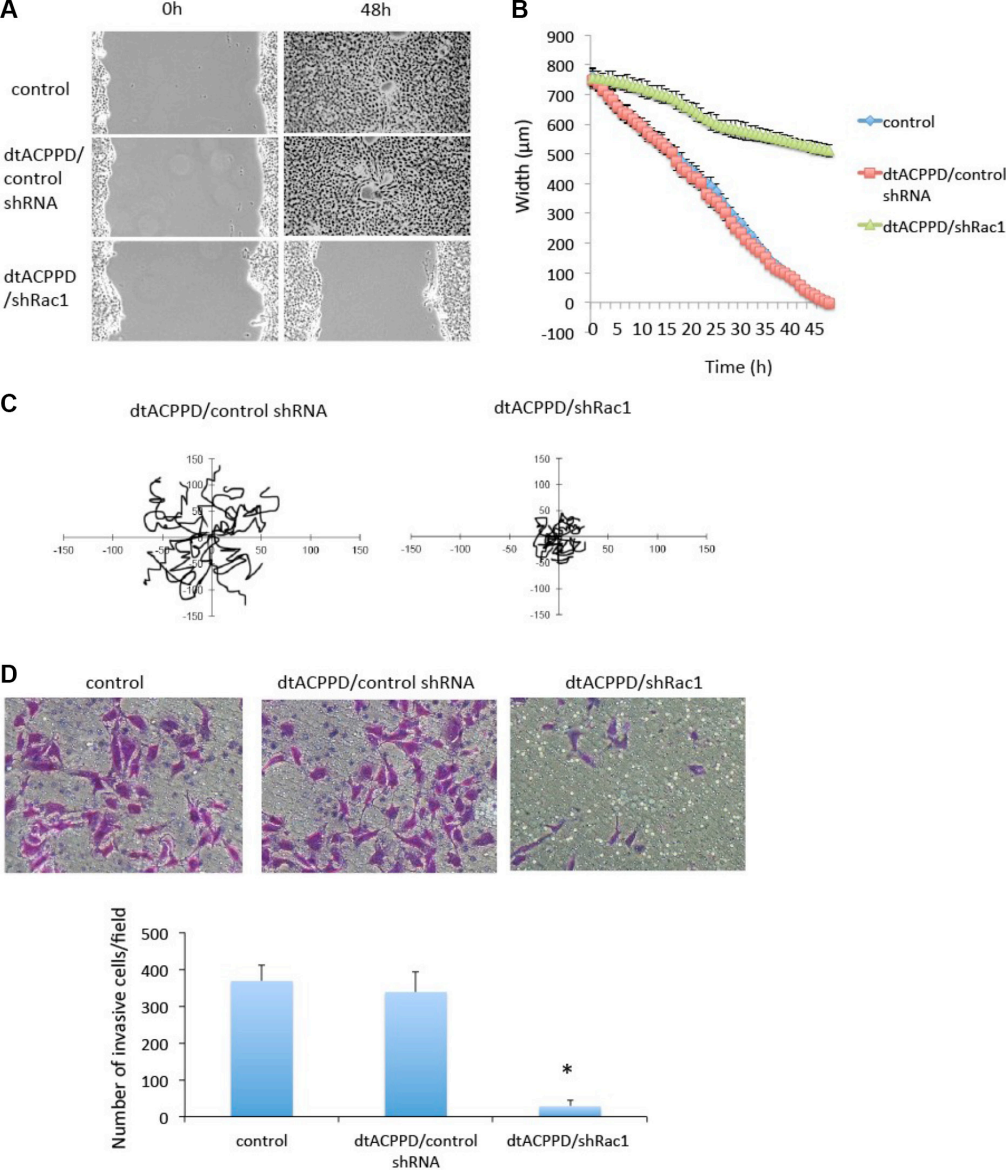
Motility of colorectal cancer cells after treatment with dtACPPD /shRNA nanoparticles (**A**) Cell migration ability after different treatments was tested by a wound-healing assay in HCT116 cells. (**B**) Quantification from Cell-IQ showing wound width as the mean with SD for percent closure of the original wound in triplicate plates. Similar results were obtained in three experiments. (**C**) Treated cells were plated sparsely and monitored by time-lapse microscopy for 16 h. The migration of cells was tracked from 15 random cells. (**D**) The invasive ability of cells was tested by an invasion assay. Invading cells were quantified.

Given the fact that dtACPPD/shRac1 nanoparticles can target and accumulate in tumor cells, we next studied whether dtACPPD/shRac1 nanoparticles affect cellular features that are crucial for metastasis of cancer cells. HCT-116 cells were incubated with dtACPPD/shRac1 for 48 h at pH 6.8, and cell migration was investigated by scratch assay and evaluated by Cell-IQ system. Figure 4A and 4B showed that dtACPPD/shRac1 significantly blocked cell migration compared with empty vector nanoparticles and control untreated cells. Cell migration was tracked by random migration analysis. Figure 4C showed that cells moved in a directed manner when incubated with empty nanoparticles; on the other hand, cells moved randomly and lost polarity when incubated with dtACPPD/shRac1, which can significantly abolish directional migration in cells. Moreover, dtACPPD/shRac1 strikingly blocked HCT-116 invasive ability (Figure 4D). These results suggested dtACPPD/shRac1 nanoparticles are capable of inhibiting colon cancer cell motility and invasion.

### Regulation of dtACPPD/shRac1 nanoparticles on MMPs and EMT in colon cancer cells

**Figure 5:**
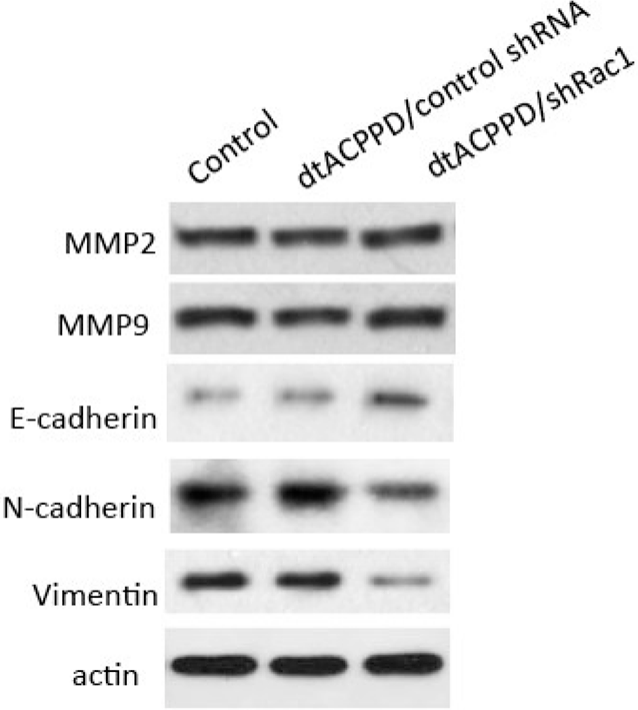
Effects of dtACPPD/shRNA nanoparticles on metastasis and EMT related markers HCT116 cells were treated by atCDDP/shRNA nanoparticles for 24 h. Expression of indicated markers was detected by corresponding antibodies.

To undergo invasion, cancer cells need to produce enzymes, such as MMPs, which play a role in digesting cell adhesion molecules and ECM proteins, [20]. We next assessed the expression of MMP2 and MMP9 in HCT-116 cells. Figure 5 showed that dtACPPD/shRac1 incubation did not alter MMP2 and MMP9 expression in cells, which potentially suppressed the breakdown of the surrounding of tissue. The epithelial-to-mesenchymal transition (EMT) enhances the mobility of cancer cells, facilitating their distant metastasis [21], showing suppression of epithelial markers, such as E-cadherin, as well as the overexpression of mesenchymal markers, such as N-cadherin and Vimentin. In our study, E-cadherin expression in HCT-116 cells at pH of 6.8 was elevated by incubation of dtACPPD/shRac1 nanoparticles, whereas N-cadherin and Vimentin were reduced, indicating that EMT was inhibited after silencing Rac1 by nanoparticles. Inhibition of EMT could result in retardation of metastasis of colon cancer cells.

### Cell to extracellular matrix adhesion

**Figure 6:**
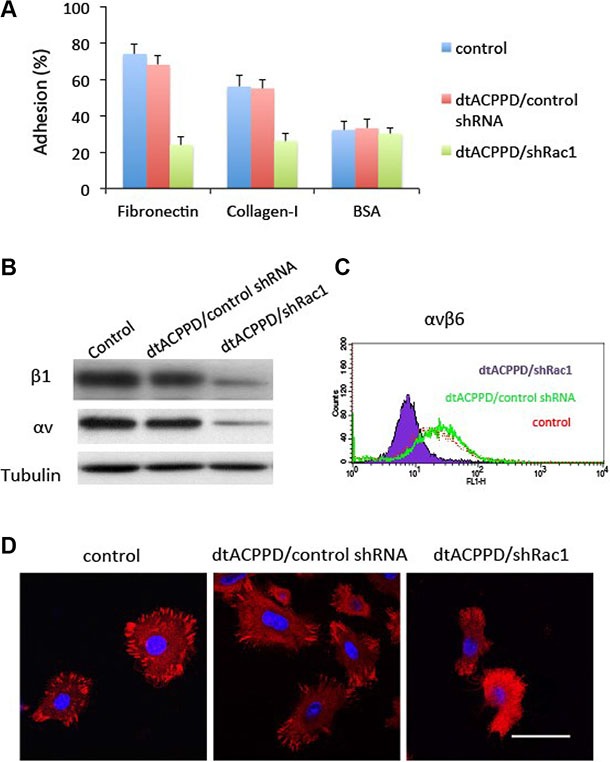
Adhesion of colorectal cancer cells after treatments with dtACPPD/shRNA nanoparticles (**A**) Adhesion assay comparing HCT116 cell adhesion to fibronectin, collagen I or BSA (negative control) coated wells. (**B**) Cell lysates were immunoblotted with antibodies against indicated proteins. (**C**) αvβ6 expression in HCT116 cells was tested by flow cytometry. (**D**) Focal adhesion was detected by immunofluorescent staining using an antibody against FAK (red). DAPI was used to stain nuclei. Scale bar is 20 μm.

Metastatic cancer cells needs to establish new contact points between a cell and its underlying substrate ECM to provide the necessary adhesion required to move forward. Therefore, cell to ECM adhesion is a key step when cancer cells plant themselves in distant locations during metastasis [22]. Regulation of cell to ECM adhesion may play important roles in cell motility. Pasapera reported that activated Rac1 promoted focal adhesion and thereby enhanced cell migration. In our study, dtACPPD/shRac1 nanoparticles were effective in impeding the adhesive potential of colon cancer cells to matrix substrates, fibronectin and collagen-I compared to BSA as a negative control (Figure 6A). The dtACPPD/shRac1 nanoparticles down-regulated the expression of integrin αv and β1 (Figure 6B), which are crucial for cancer cells to ECM adhesion [23]. Flow cytometry results indicated that dtACPPD/shRac1 nanoparticles also decreased integrin αvβ6, which is important for cell to ECM adhesion and metastasis of colon cancer [24].

Figure 6B, 6C showed that dtACPPD/shRac1 nanoparticles markedly reduced focal adhesion in HCT116 cells. Meanwhile, FAK expression was suppressed when cells were incubated with dtACPPD/shRac1 nanoparticles, which could be the reason underlying the reduced focal adhesion. Suppression of Rac1 expression inhibited FAK expression, resulting in blocked focal adhesion in HCT116 cells (Figure 6D). This could reduce the adhesive ability to remote organs for colorectal cancer cells.

**Figure 7:**
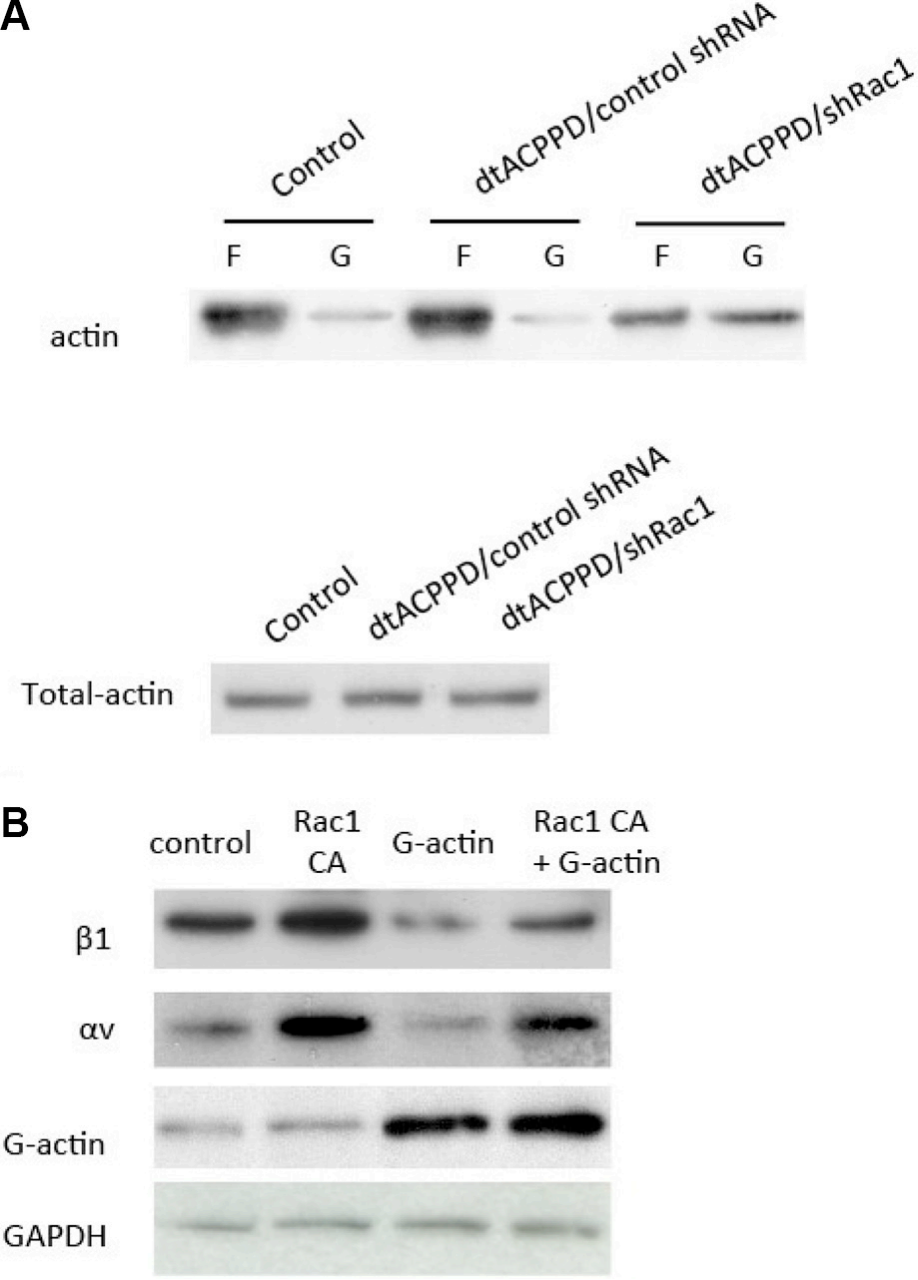
Rac1 regulates cell to matrix adhesion via cytoskeletal reorganization (**A**) F-actin and G-actin fractions were prepared and subjected to Western blotting. (**B**) HCT116 cells were transfected with control plasmid, Rac1 CA (constitutive active Rac1), incubated with additional G-actin, or Rac1 CA and G-actin. Cell lysates were immunoblotted with antibodies against the indicated proteins.

We next proceeded to determine the mechanism by which Rac1 regulates cell to matrix organization. Rac1 is important for filament dynamics and cytoskeletal reorganization. Cytoskeletal organization is a process of depolymerization of filamentous actin (F-actin) and polymerization of G-actin. We found that incubation of dtACPPD/shRac1 nanoparticles dramatically increased G-actin content, suggesting the enhanced depolymerization of actin (Figure 7A). To further confirm whether the increased content of G-actin is responsible for cell to matrix adhesion, we then enhanced Rac1 expression by Rac1 CA plasmid with or without addition of pure G-actin (whose polymerization was blocked by latrunculin B). The results showed that overexpression of Rac1 elevated the integrin αv and β1 levels (Figure 7B), corresponding to higher cell to matrix adhesion (Figure 7C). However, additional incubation with G-actin markedly downregulated integrin αv and β1 levels, corresponding to lower cell to matrix adhesion. This alteration could be partially impaired by overexpression of Rac1.

### dtACPPD/shRac1 nanoparticles reduced hepatic metastasis of colon cancer *in vivo*

**Figure 8:**
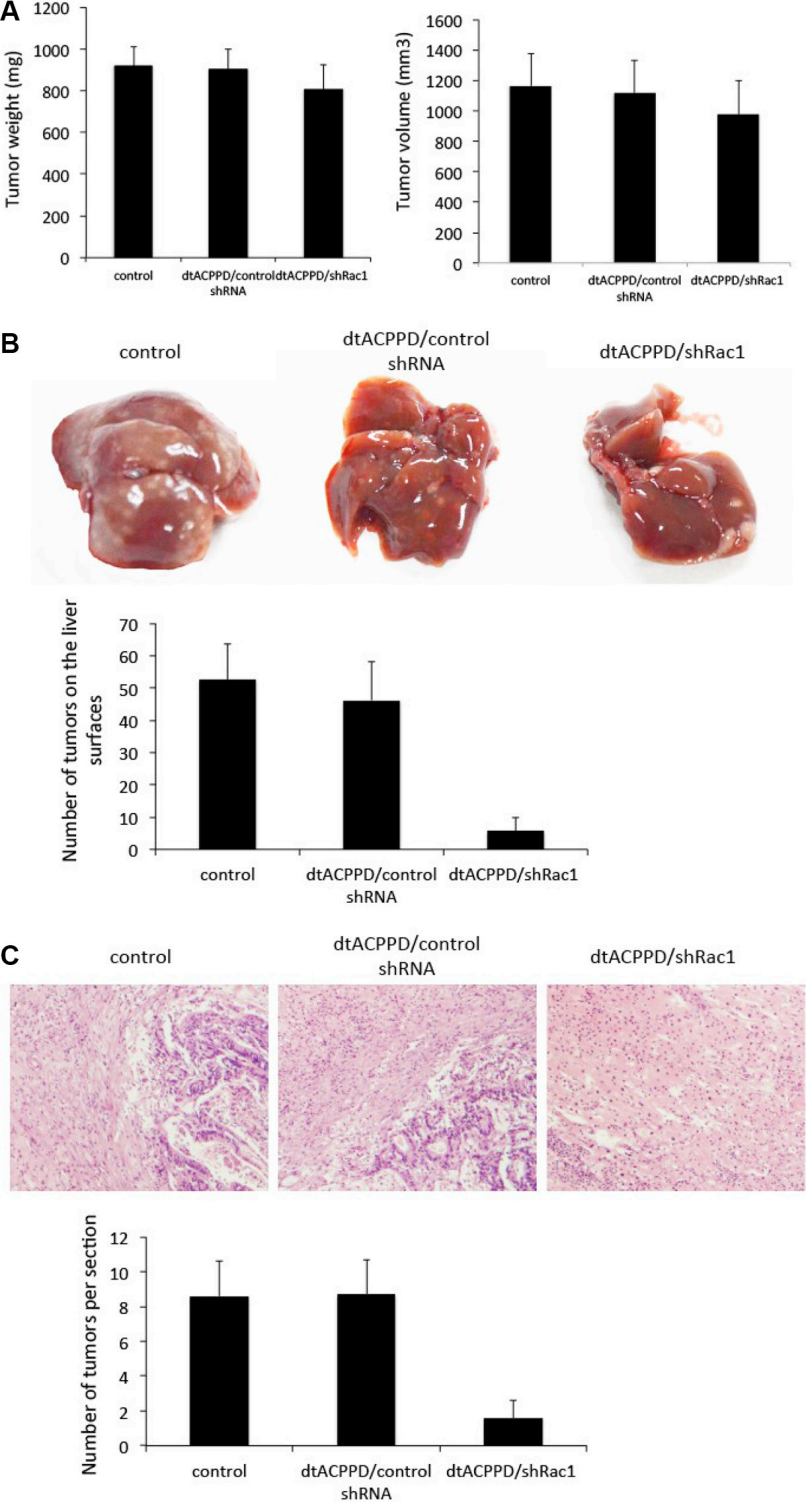
dtACPPD /shRNA nanoparticles inhibited the growth of colon cancer xenografts and liver metastasis in nude mice (**A**) The weight and volume of tumors at the end of drug treatment. The drugs were administered 3 times/week for 3 successive weeks. (**B**) Representative livers at the end of drug treatment. The number of tumors on liver surfaces were calculated. (**C**) Representative typical histopathological images of liver (H&E staining, × 400 magnification).

To evaluate the potential efficiency of dtACPPD/shRac1 on final metastatic cancer, mice were injected subcutaneously with HCT116 cells, until tumors grew bigger than 1000 mm^3^ before dtACPPD/shRac1 was administrated to the mice. The metastatic lesions were found in the livers when tumors reached this size. dtACPPD/shRac1 was then given to the mice 3 times a week for 3 weeks after the 21st day post injection of cells. The treatment with dtACPPD/shRac1 showed slightly inhibited the primary tumor growth (Figure 8A). However, dtACPPD/shRac1 treatment led to a significant decrease in the number of tumor nodules observed on the liver surface (Figure 8B). HE staining showed that both the size and number of tumors inside the liver were reduced in dtACPPD/shRac1-treated mice (Figure 8C). These results confirmed that dtACPPD/shRac1 nanoparticles significantly inhibit tumor metastasis *in vivo*.

## DISCUSSION

As a key molecule that regulates cytoskeleton reorganization, Rac1 is crucial for cell motility [7]. Mounting evidence showed that Rac1 expression and activity are both increased in many cancer types, including colon cancer and it enhanced the metastasis in cells [18]. In this study, we found that Rac1 was related to the metastasis and survival time of colon cancer. Therefore, inhibition of Rac1 specifically in cancer cells would be a strategy to retard metastasis. As to undergo invasion, cancer cells need to produce enzymes, such as MMPs, to degrade cell adhesion molecules and ECM proteins. We further demonstrated that MMP2 was high expressed in colon cancer cells. This gave us a target to recognize colon cancer cells. We therefore used the dtACPPD system that was designed to recognize MMP2 in cancer cells, by means of it to target cancer cells. Another advantage of this system is that it is easier to deliver genes into cells with lower pH environment, such as cancer cells. Indeed, Figures 3 and 4 showed that dtACPPD/shRac1 was expressed in cancer cells *in vitro* or *in vivo*, suggesting that dtACPPD/shRac1 can target colon cancer cells. Moreover, the knock down efficiency was satisfactory using this system. On the other hand, dtACPPD/shRac1was also found distribute in vital organs such as heart, liver, kidney and spleen as shown in Figure 3. This remained a concern of toxic effects of dtACPPD/shRac1 nanoparticles. In the 3-week treatment of colon cancer xenograft in mice, we did not find obvious pathological phenomena in vital organs (data not shown). These demonstrated that dtACPPD/shRac1 nanoparticles are effective and safe to use.

Cancer cells mobility was decided by complex effectors, such as cytoskeleton organization, cell to cell adhesion, cell to matrix adhesion, etc. EMT plays a crucial role in cancer cells metastasis. Although dtACPPD/shRac1 nano-particles did not affect MMP2 and MMP9 expression unexpectly, it effectively inhibited cells migration and invasion as shown in Figure 4. This could due to the impairment of cytoskeleton reorganization (Figures 3 and 7) and inhibition of EMT in colon cancer cells after treatment of dtACPPD/shRac1 nano-particles (Figure 5).

The process of cancer metastasis is related to increased cell invasion and cellular focal adhesion. FAK, a cytoplasmic tyrosine kinase, plays central roles in regulating focal adhesion formation, actin-myosin dynamics, EMT, migration, and invasion [25], highlighting its importance in tumor progression and metastasis. The increased expression of FAK has been found in several types of human cancer and was associated with the enhanced metastasis of various solid tumors and poor prognosis [26, 27]. Moreover, FAK signaling regulates cancer growth and metastasis [28]. dtACPPD/shRac1 nanoparticles markedly reduced cell to extracellular matrix adhesion and focal adhesion in HCT116 cells (Figure 6). Meanwhile, FAK expression was suppressed when cells were incubated with dtACPPD/shRac1 nanoparticles, which could be the reason underlying the reduced focal adhesion. This could reduce the adhesive ability to remote organs for colorectal cancer cells, thereby reduce the anchor and growth of tumor.

Due to above reasons, administration of dtACPPD/shRac1 nanoparticles inhibited colon cancer metastasis into liver. Using dtACPPD/shRac1 system to deplete Rac1 expression in colorectal cancer cells markedly inhibited cells migration and invasion *in vitro*, as well as metastasis *in vivo* by impairing cytoskeleton reorganization and adhesion of cell-to-extracellular matrix. dtACPPD/shRac1 nanoparticles showed a potential usage on treat advance colorectal cancer.

## MATERIALS AND METHODS

### Materials

atCDDP was synthesized according to Huang et al. EGFP-tagged shRNA plasmid and constitutive active Rac1 (Rac1 CA) plasmid were purchased from Genepharm. Co. Ltd. The colorectal cancer cell lines HCT116 were purchased from ATCC (Middlesex, UK). DMEM medium and fetal calf serum (FCS) were supplied by Lonza (Wokingham, UK).

### Preparation of nanoparticles

The freshly prepared nanocarrier was diluted to an appropriate concentration, and then the shRNA (100 μg DNA/mL in 50 mM sodium sulfate solution) was added and immediately vortexed for 30 s. The complex was then exposed to UV light (365 nm) for 1 h, followed by precipitation by means of adding ethanol at a final concentration of 30% (v/v). The precipitate was collected by centrifugation and re-dissolved in 50 mM sodium sulfate solution.

### Nanoparticle characterization

The size distribution and zeta potential of dtACPPD /shRNA were measured using a Zetasizer (ZS90, Malvern, U.K.) with a scatter angle of 90° at 25°C. The morphology of nanoparticles was observed by scanning electron microscopy (SEM) (JEOL JSM T330A).

### Tissue collection

Fresh tissue samples of 66 colon cancers with hepatic metastasis tumor tissues were collected from surgical resections and endoscopic biopsies in the First Affiliated Hospital, Huzhou University (Huzhou, China). All tissues were stored at −80°C. All samples were obtained from patients who gave informed consent to use excess pathological specimens for research purposes. All tissues had a definitive pathological diagnosis. The protocols used in the study were approved by the Hospital's Protection of Human Subjects Committee. The use of human tissues was approved by the Institutional Review Board of the Wenzhou Medical University and was performed in accordance with international guidelines for the use of human tissues.

### Colorectal cancer xenograft experiments

Five-week-old female BALB/c Nu/Nu athymic nude mice (Biotechology & Cell Biology Shanghai, China) were housed under pathogen-free conditions according to Wenzhou Medical University animal care guidelines, and the animal experiments were reviewed and approved by the Ethical Committee. HCT116 cells (5 × 10^5^) were subcutaneously injected. After 3 weeks, the tumor bearing mice were randomly subdivided into 3 groups (8 mice/group): control; dtACPPD /control shRNA (50 μg DNA); and dtACPPD /shRac1 (50 μg DNA). The animals were treated 3 times/week (i.v) for successive 3 weeks. At the end of the experiments, the animals were sacrificed. The tumors were removed, photographed and subjected to further analysis. The tumor volume was calculated by the following formula: V = (L × W^2^) × 0.5, where L is the length and W is the width of the tumor.

### *In vivo* distribution and gene transfection

At the 21st day after implantation, tumor-bearing mice were injected intravenously through the tail vein with 200 μL dtACPPD/ DNA at a dose of 50 μg DNA. Then, 24 h after administration, the mice were anesthetized and visualized by a Cambridge Research & Instrumentation *in vivo* imaging system (CRi, MA, USA). After that, mice were sacrificed and the main organs (heart, liver, spleen, lung, and kidneys) were excised carefully to compare the relative accumulation.

### Western blot

Rac1 expression was tested using western blot according to a standard protocol [7]. In brief, lysates from cells or tumor tissues were prepared. Protein was resolved by SDS-PAGE and transferred to PVDF membrane, and immunoblot analysis was performed. The following antibodies were applied overnight at 4°C: anti-Rac1 (1:1,000 dilution) and mouse anti-actin (1:50,000). All of antibodies were purchased from Cell Singlling Technology Co. Blots were than incubated with secondary horseradish peroxidase–conjugated IgG and visualized with enhanced chemiluminescence reagents.

### Wound healing assay

Cell migration was analyzed by a wound healing assay. Cells were grown to confluency and starved overnight. Then, a scratch wound in the monolayer was performed by dragging a 1-ml pipette tip across the layer. Cells were cultured as described above, and wound closure was followed by microscopy at 0 h and 48 h after wound infliction. Experiments were repeated three times.

### Invasion assay

As described previously [14], briefly, 3 × 10^4^ cells in serum free DMEM were seeded into the upper chambers of 8-μM pore Transwells. For assay of invasion through a Matrigel barrier, cells were allowed to migrate for 36 h. Migrated cells were fixed, stained, counted from six random fields and averaged. Experiments were repeated three times.

### Random migration analysis

HCT116 cells were plated sparsely (3 × 10^4^ cells) and treated following a previous report [15]. Cells were incubated overnight in culture medium at 37°C and 5% CO_2_. Phase-contrast images were taken every 15 min on a Widefield CCD system (Carl Zeiss MicroImaging, Inc.). Tracks of individual cells were analyzed using ImageJ software (National Institutes of Health, Bethesda, MD, USA).

### Immunofluorescence

In brief, cells were washed with PBS, fixed in 4% paraformaldehyde, permeabilized with 0.1% Triton X-100, and blocked in 2% BSA before staining with anti-phalloidin and anti-FAK antibodies (both from Cell Signaling). Nuclear DNA was visualized with 4′, 6-diamidino-2-phenylindole (DAPI) (Sigma). Images were collected with a confocal microscope (Leica Microsystems).

### Cell adhesion assay

As described previously [16], prior to plating the cells, 96 well plates were coated with matrix proteins (fibronectin, 1 μg/well; collagen 1, 10 μg/well; and BSA, 1 μg/well, all from Sigma) and dried at room temperature. HCT116 cells with or without dtACPPD /shRNA treatment were added to each well and incubated at 37°C for 1 h. PBS was added to remove non-adherent cells. Then, adhesive cells were fixed with 3% formaldehyde and then stained with 0.1% crystal violet (038-17792; Wako Pure Chemical Industries, Ltd., Osaka, Japan). After washing with PBS, 10% acetic acid was added, and the absorbance at 570 nm was determined using an ELISA plate reader (iMark^™^ Microplate Reader; Bio-Rad, Hercules, CA, USA).

### G-actin purification

Actin was purified from rabbit muscle as described [17], dialyzed against G buffer (2 mM Tris pH 8, 0.2 mM ATP, 0.5 mM DTT, 0.1 mM CaCl2, 1 mM NaN3), blocked from further polymerization by incubation with a fivefold molar excess of Latrunculin B (#428020, Calbiochem), and used in biochemical and structural studies.

### Cellular F-actin/G-actin assays

F-actin and globular actin (G-actin) fractions were obtained using an F-actin/G-actin assay kit (BK 037, Cytoskeleton).

### H&E staining

Paraffin-embedded liver slides were deparaffinized, hydrated, and then stained with hematoxylin for 1 minute. After rinsing, the slides were stained with eosin for 1 minute, followed by more rinsing, and coverslips were mounted onto slides with Permount (Fisher Sci, Loughborough, UK).

### Statistical analysis

The statistical significance of treatment outcomes was assessed using the Student's *t*-test; *p* < 0.05 was considered statistically significant in all analyses.
